# Integrated Pest Management: An Update on the Sustainability Approach to Crop Protection

**DOI:** 10.1021/acsomega.4c06628

**Published:** 2024-09-28

**Authors:** Wentao Zhou, Yashwanth Arcot, Raul F. Medina, Julio Bernal, Luis Cisneros-Zevallos, Mustafa E. S. Akbulut

**Affiliations:** † Artie McFerrin Department of Chemical Engineering, Texas A&M University, College Station, Texas 77843, United States; ‡ Department of Entomology, Texas A&M University, College Station, Texas 77843, United States; § Department of Horticultural Sciences, Texas A&M University, College Station, Texas 77843, United States; ∥ Materials Science and Engineering, Texas A&M University, College Station, Texas 77843, United States

## Abstract

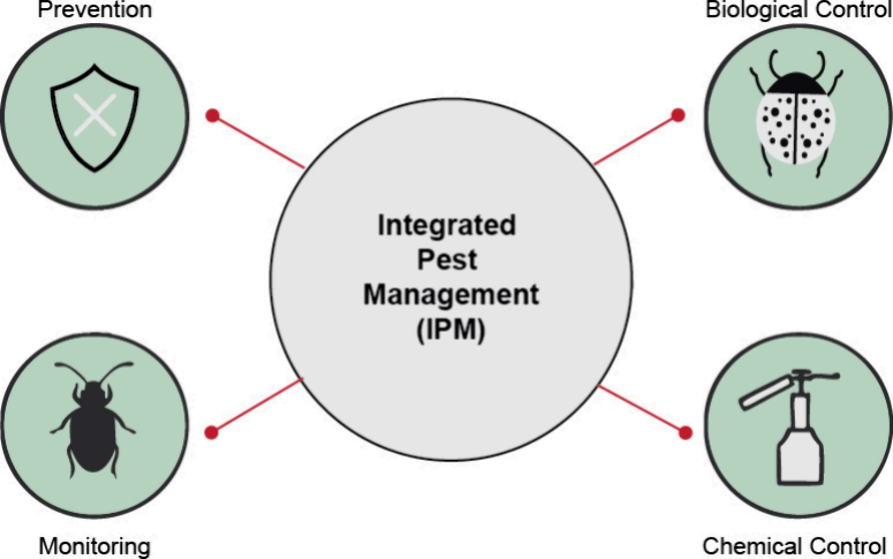

Integrated Pest Management (IPM) emerged as a pest control framework promoting sustainable intensification of agriculture, by adopting a combined strategy to reduce reliance on chemical pesticides while improving crop productivity and ecosystem health. This critical review synthesizes the most recent advances in IPM research and practice, mostly focusing on studies published within the past five years. The Review discusses the key components of IPM, including cultural practices, biological control, genetic pest control, and targeted pesticide application, with a particular emphasis on the significant advancements made in biological control and targeted pesticide delivery systems. Recent findings highlight the growing importance of genetic control and conservation biological control, which involves the management of agricultural landscapes to promote natural enemy populations. Furthermore, the recent discovery of novel biopesticides, including microbial agents and plant-derived compounds, has expanded the arsenal of tools available for eco-friendly pest management. Substantial progress has recently also been made in the development of targeted pesticide delivery systems, such as nanoemulsions and controlled-release formulations, which can minimize the environmental impact of pesticides while maintaining their efficacy. The Review also analyzes the environmental, economic, and social dimensions of IPM adoption, showcasing its potential to promote biodiversity conservation and ensure food safety. Case studies from various agroecological contexts demonstrate the successful implementation of IPM programs, highlighting the importance of participatory approaches and effective knowledge exchange among stakeholders. The Review also identifies the main challenges and opportunities for the widespread adoption of IPM, including the need for transdisciplinary research, capacity building, and policy support. In conclusion, this critical review discusses the essential role of IPM components in achieving the sustainable intensification of agriculture, as it seeks to optimize crop production while minimizing adverse environmental impacts and enhancing the resilience of agricultural systems to global challenges such as climate change and biodiversity loss.

## I Introduction

Integrated Pest Management (IPM), which is a thorough, ecological approach to managing pests in agricultural systems, involves the strategic integration of multiple control methods, including cultural, biological, and chemical tactics, to maintain pest populations below economically damaging levels while minimizing risks to the environment and public health.^1^ IPM emphasizes the employment of preventive measures, monitoring, and decision-making based on established thresholds, rather than relying solely on reactive pesticide applications.^2,3^ The fundamental principles of IPM include preventing pest problems through cultural practices, such as crop rotation and sanitation; monitoring pest populations and their natural enemies; using economic thresholds to guide management decisions; employing a blend of biological, physical, and chemical control methods; and evaluating the effectiveness of interventions.^4,5^ Through the incorporation of multidisciplinary knowledge and a systems-based approach, IPM is aimed at optimizing agricultural productivity while preserving ecosystem services and mitigating the detrimental consequences of conventional pesticide reliance.^6,7^

Sustainable pest control practices, within an IPM framework, are important for addressing the challenges posed by the increasing global demand for food, the necessity to conserve national bioresources, and the urgency to mitigate the adverse effects of climate change.^8,9^ Conventional pest control practices, characterized by the intensive application of pesticides, have caused a multitude of ecological, economic, and societal challenges. These include the appearance of pesticide resistance, the disruption of beneficial arthropod communities, the contamination of soil and water, and the potential exposure of agricultural workers and consumers to hazardous chemicals.^10,11^ Conversely, IPM presents a more sustainable model for pest control by restricting pesticide treatment to economically and ecologically justifiable thresholds. By curtailing overall chemical pesticide reliance, IPM fosters biodiversity conservation, safeguards ecosystem services, and strengthens the stability of agricultural systems. Furthermore, the utilization of IPM procedures can yield economic benefits for farmers through reduced input costs and enhanced crop yields, whereas simultaneously promoting food safety and produce quality for consumers.^12^

In essence, given the pressing necessity to develop more sustainable and robust agricultural frameworks in the light of mounting global challenges, the widespread embracing of IPM practices is essential.^13,14^ IPM not only addresses the direct impacts of pests on crop production but also backs the broader objectives of sustainable development, including the conservation of natural resources, the protection of public health, and the promotion of social and economic well-being.

This review mostly presents the most recent advances in IPM in the past five years after briefly summarizing the traditional IPM basics. It is organized in several sections including recent updates on IPM components (i.e., cultural, monitoring, biological and chemical), sustainability benefits of IPM (i.e., environmental, economic and social), challenges and opportunities, research gaps and priorities and an overall summary of the review outcome.

## II Components of IPM

**Figure 1:**
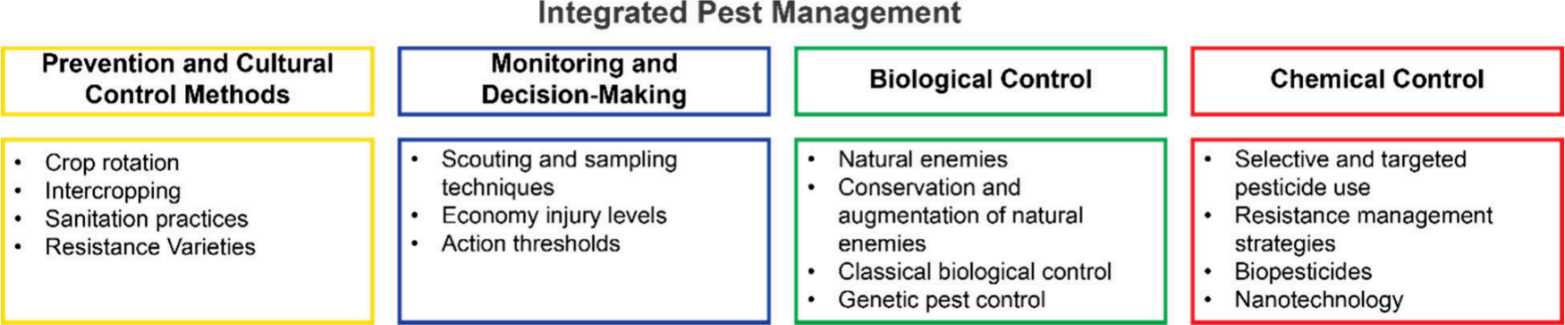
Key components of an Integrated Pest Management (IPM) program.

Figure 1 illustrates the key components of an IPM program, which is a thorough approach to managing pests in an environmentally and economically sustainable manner. As shown in the figure, IPM relies upon a blend of strategies, including prevention and cultural control methods, monitoring and decision-making tools, biological control, and chemical control. Prevention and cultural control methods involve methods such as sanitation, crop rotation, intercropping, and the utilization of resistant varieties to create conditions that are less favorable for pest populations to develop. Monitoring and decision-making tools (i.e., economic injury levels, action thresholds, scouting, and sampling techniques) help farmers to assess pest populations and determine when intervention is necessary. Biological control methods, including the employment of natural enemies, conservation and augmentation of beneficial insects, genetic control, and classical biological control (CBC), harness the power of predators/parasites to keep pest populations in check. Lastly, chemical inactivation methods, including biopesticides, selective/targeted pesticide utilization, and nanotechnology, are used judiciously to control pests when other methods are inadequate. By integrating these diverse strategies, IPM can successfully manage pests by shrinking risks to public health and the environment.

### II.A Prevention and Cultural Control Methods

Crop rotation, a cornerstone of preventive pest management within the IPM framework, entails the sequential cultivation of dissimilar crops on a given field across multiple growing seasons.^15^ The efficacy of crop rotation for suppressing pest populations is attributable to several mechanisms, including the spatiotemporal separation of host crops, the incorporation of nonhost crops that function as barriers or trap crops, and the fostering of beneficial beings via enhanced biodiversity.^16^ The effectiveness of crop rotation as a IPM strategy is contingent upon the judicious selection and organization of crops in a temporal sequence, the diversity of crops included in the rotation scheme, the length of the rotation cycle, and the strategic incorporation of cover crops or green manures.^15^ These factors should be carefully evaluated and optimized to maximize the potential benefits of crop rotation in suppressing pest populations and mitigating the associated crop damage.^17^ The alternation of nonhost crops, exemplified by cereals, with host crops, such as vegetables, in a strategic rotational sequence has been demonstrated to effectively mitigate the incidence and severity of soil-borne phytopathogens and plant-parasitic nematodes across a diverse array of crops, as corroborated by prior publications.^18^ Similarly, the inclusion of leguminous crops in rotation can suppress weed populations through allelopathic effects and competition for resources, while also ameliorating soil condition and fertility.^19^

Intercropping, an effective cultural control strategy, refers to the concurrent cultivation of multiple crop species within a single field.^20^ This prophylactic practice utilizes ecological interactions between diverse plant species to establish agroecosystems less conducive to pest proliferation while simultaneously fostering the activity of natural enemies. The mechanisms beyond the pest-suppressive effects of intercropping are complex, encompassing factors including resource competition, physical barriers, allelopathy, and habitat manipulation.^21^ The efficacy of intercropping as a pest management strategy is contingent upon the judicious selection of companion crops, their precise spatial configuration, and the ideal timing of their establishment.^22,23^ For instance, the utilization of aromatic plants, exemplified by *Ocimum basilicum* or *Mentha* spp., as intercrops were reported to repel or mask the volatile olfactory cues exploited by pests to locate their host plants, thereby diminishing pest infestation levels.^24,25^ Aside from its direct impact on pest populations, intercropping can also boost the overall resilience and yield of agroecosystems by augmenting soil fertility, optimizing water use efficiency, and mitigating the risk for crop failure stemming from abiotic stressors.^26−28^

Sanitation practices, are cultural control practices that involve the removal and destruction of pest-infested plant material, crop residues, and other sources of pest inoculum from farmlands and surrounding areas.^29^ These practices aim to shrink emergent pest populations and prevent their spread within and between cropping seasons, thus minimizing the necessity for curative interventions.^30^ For example, the elimination of fallen fruits and mummified nuts in almond orchards were demonstrated to significantly reduce the overwintering population of navel orangeworm (*Amyelois transitella*), a main pest of almonds and other tree nuts.^31^ Aside from these field-level measures, sanitation practices also encompass the cleaning and disinfection of farm equipment, storage facilities, and transportation vehicles to prevent the introduction and spread of pests from external sources.^32^

The employment of resistant varieties is a fundamental cultural control strategy that exploits the genetic diversity of crops to minimize the adverse-effects of pests and diseases on crop production.^33^ The use of resistant varieties in IPM programs aims to decline the dependence on pesticides, minimize yield losses, and amend the overall sustainability and resilience of crops. The mechanisms of resistance in crop plants are diverse and can be divided into three main categories: antixenosis, antibiosis, and tolerance.^34^ Antixenosis renders the plant less attractive or suitable for feeding, oviposition, or shelter. Antibiosis involves plant characteristics that have direct adverse consequences on pest growth, development, reproduction, or survival, such as the production of toxic secondary metabolites or of physical barriers. Tolerance describes the capacity of a plant to withstand or recover from pest damage without significant yield reduction, often through compensatory growth or enhanced stress responses. Using Bt cotton varieties, which express insecticidal proteins from the bacterium *Bacillus thuringiensis*, has been a major success story in IPM, leading to significant falls in pesticide utilization and improved control of lepidopteran pests, such as cotton bollworm.^35^ However, the prevalent adoption of Bt cotton has also raised concerns about the probability for resistance development in pest populations, highlighting the necessity for effective resistance management strategies.^36,37^

### II.B Monitoring and Decision Making

Scouting and sampling techniques are fundamental components of the monitoring and decision-making process in IPM programs.^38,39^ Aside from these sampling designs, various tools and techniques are employed to monitor pest populaces and their negative-effects on crop plants, including visual inspection, the employment of sweep nets, sticky traps, pheromone traps, and remote sensing technologies.^40,41^ Remote sensing techniques, such as aerial photography, satellite imagery, and unmanned aerial vehicles (UAVs), are increasingly being used to monitor crop health and detect pest outbreaks over large spatial scales.^42−44^ The integration of diverse monitoring tools and techniques, coupled with appropriate sampling designs, empowers IPM practitioners to make data-driven decisions regarding the necessity and timing of pest management interventions. Recently, the advancement of artificial intelligence (AI) for identification and decision-making has been utilized in IPM: Batz et al.^45^ reported the several ways for AI to enhance aphid pest forecasting: 1) identification of insects best on image recognition and Deep Learning, 2) forecasting model based on Machine Learning and neural networks and 3) optimizing the monitoring infrastructure to improve predictive models. Ali et al.^46^ reported an artificial intelligence (AI)-enabled IoT-based pests detection method employing pest sound analytics in large agricultural area and among the four models mentioned in their work, the CNN-Bi-LSTM model could achieve 98.91% accuracy. However, the usage of AI has been more frequently in not only IPM but also the whole agriculture area, there are still several obstacles restricting AI decision support systems: AI technology effectiveness, functionality under field conditions, the level of computational expertise and power required to use and run the system and system mobility.^47^

Economic injury levels (EILs) and action thresholds (ATs) are essential tools in IPM decision-making.^48−50^ They help farmers and pest management professionals determine when pest control measures are economically warranted. EILs represent the pest population density at which the cost of crop damage equals the cost of control, while ATs are set at a lower pest density to prevent populations from reaching the EIL.^48−50^ By considering pest density, crop susceptibility, and the costs and benefits of different management strategies; EILs and ATs enable the implementation of timely and cost-effective pest control interventions.^51−53^ This approach minimizes redundant pesticide applications, reducing the ecological impact and economic burden accompanying pest management. The calculation of EILs and ATs requires extensive information on the autoecology or natural history of the pest species, the crop, and their interactions aside from the economic factors influencing the cost-benefit analysis of pest management decisions.^54^ For instance, the EIL for soybean aphid (*Aphis glycines*) in soybean production has been estimated at 674 aphids per plant, based upon the relationship between aphid density, soybean yield, and the cost and efficacy of insecticide treatments.^55−57^ The corresponding AT for soybean aphid is set at 250 aphids per plant, providing a buffer for population growth and allowing time for the implementation of control measures to take effect.^55−57^ The efficacious implementation of EILs and ATs within IPM presents some challenges, including the necessity for accurate and timely monitoring data, the variability in pest damage relationships across diverse environmental conditions and crop phenological stages, and the propensity for multispecies pest interactions that can synergistically impact crop yield.^58,59^

### II.C Biological Control

Natural enemies, encompassing parasitoids, predators, and pathogens, constitute an important component of biological pest control within IPM programs.^60−62^ Such beneficial organisms can enable the regulation of pest populaces through diverse mechanisms, including direct predation, parasitism, and infection, frequently maintaining pest densities below economically damaging thresholds.^63−65^ The prolific integration of natural enemies into IPM necessitates a comprehensive elucidation of their biology and ecology with both target pests and the crop environment.^66^ Predators, encompassing a diverse range of species from arthropods (e.g., ladybird beetles, lacewings, spiders) to vertebrates (e.g., birds, rodents), actively hunt and prey upon multiple individuals throughout their life cycle.^67−69^ The influence of predators on pest populations depends on their feeding rate, functional response, numerical response, prey preference, and several other ecological components.^70^ Parasitoids, instead, are insects that lay their eggs or/in a host individual, eventually eliminating the host while the parasitoid larvae develop.^71,72^ Pathogens, including viruses, bacteria, fungi, and nematodes, infecting and causing disease in pest populations, leading to reduced growth, reproduction, and survival.^73^

Classical biological control (CBC) is a strategy that falls within the broader umbrella of IPM and biological control, which entails the importation and establishment of natural adversaries sourced from the native selection of the target pest.^74,75^ This strategy seeks to attain long-term, sustainable pest suppression by reestablishing the ecological balance between the pest and its natural predators in the introduced range, thereby mitigating the adverse impacts of the invasive pest species on agroecosystems. The choice of suitable natural enemies is based on criteria such as their host specificity, climatic adaptability, reproductive potential, and searching efficiency.^65,76^ Host specificity is important to minimize the risk of nontarget effects on native species and to ensure the environmental safety of the biological control program. One of the most successful examples of CBC is the transference of some vedalia beetle (*Rodolia cardinalis*) from Australia to California in the late 19th century for the control of the cottony cushion scale (*Icerya purchasi*), a serious pest of citrus.^77^ Vedalia beetle rapidly established and spread throughout the infested areas, effectively suppressing the scale populations and saving citrus industry in California. Other notable examples involve the management of the cassava mealybug in Africa through the transference of the parasitoid wasp *Apoanagyrus lopezi* from South America.^78^ Despite these successes, CBC also faces challenges and risks, including the potential for nontarget impacts on native species, the unintended spread of introduced agents to new areas, and the possible interference with other IPM tactics.

The inclusion of natural enemies into IPM programs involves the conservation and augmentation of existing populations and the institution of new species through conservation biological control (CVBC). CVBC focuses on modifying the crop environment to favor the survival and performance of natural adversaries, such as by providing alternative food sources (e.g., pollen and nectar), shelter, and overwintering sites. Conservation and augmentation of natural predators are two key strategies within the broader framework of biological control.^60,79^ These approaches aim to enhance the abundance, diversity, and effectiveness of predators/parasitoids/pathogens in agroecosystems, thereby promoting the natural regulation of pest populations.^80^ Conservation and augmentation techniques are often utilized together with other IPM tactics, such as chemical and cultural control, to achieve sustainable and cost-effective pest management.^81^ This encompasses various practices, including the provision of alternative food sources (e.g., nectar, pollen, and alternative prey), the creation of sheltering and overwintering habitats (e.g., beetle banks and hedgerows), and the minimization of broad-spectrum pesticide applications that can adversely affect beneficial organisms.^82^ In contrast, augmentation biological control entails the cyclic release of externally grown natural adversaries to supplement existing populations or compensate for their absence.^83,84^ This approach can be further categorized into inoculative and inundative releases, contingent upon the specific goals and frequency of release events.^85,86^ The realization of augmentation biological control is contingent upon the quality/quantity of released natural enemies, the timing and frequency of releases, and compatibility with other IPM tactics. Exemplary successful augmentation programs include the distribution of the predatory mite *Phytoseiulus persimilis* to control two-spotted spider mites in greenhouse crops,^87,88^ and the release of the parasitoid wasp *Trichogramma* spp. to control lepidopteran pests in field crops.^89,90^

Gene drives have received significant attention in the field of agricultural pest control due to their potential to effectively manage or even eradicate invasive species that pose threats to crops and ecosystems.^91−93^ The ability of gene drives to rapidly spread desired traits through a population could revolutionize pest management strategies, offering a more targeted and sustainable approach compared to traditional methods such as pesticides. One promising application of gene drives in agriculture is the control of insect pests. By introducing traits that reduce the fertility or lifespan of the targeted species, gene drives could potentially suppress pest populations below economically damaging levels. For example, gene drives have been proposed as a means to combat the Asian citrus psyllid, a vector of the devastating citrus greening disease.^94^ However, the deployment of gene drives in agricultural settings also raises important ecological and ethical concerns. The potential for unintended consequences, such as the inadvertent spread of gene drives to nontarget species or the evolution of resistance in the targeted pests, requires careful consideration and robust risk assessment.^95^

### II.D Chemical Control

Among the different IPM components, perhaps chemical control is one that has experienced most novel and recent updates. Herein we include recent advances in selective and targeted pesticide use, resistance management, biopesticides and natural compounds and the use of nanotechnology.

#### II.D.1 Selective and Targeted Pesticide Use

**Figure 2:**
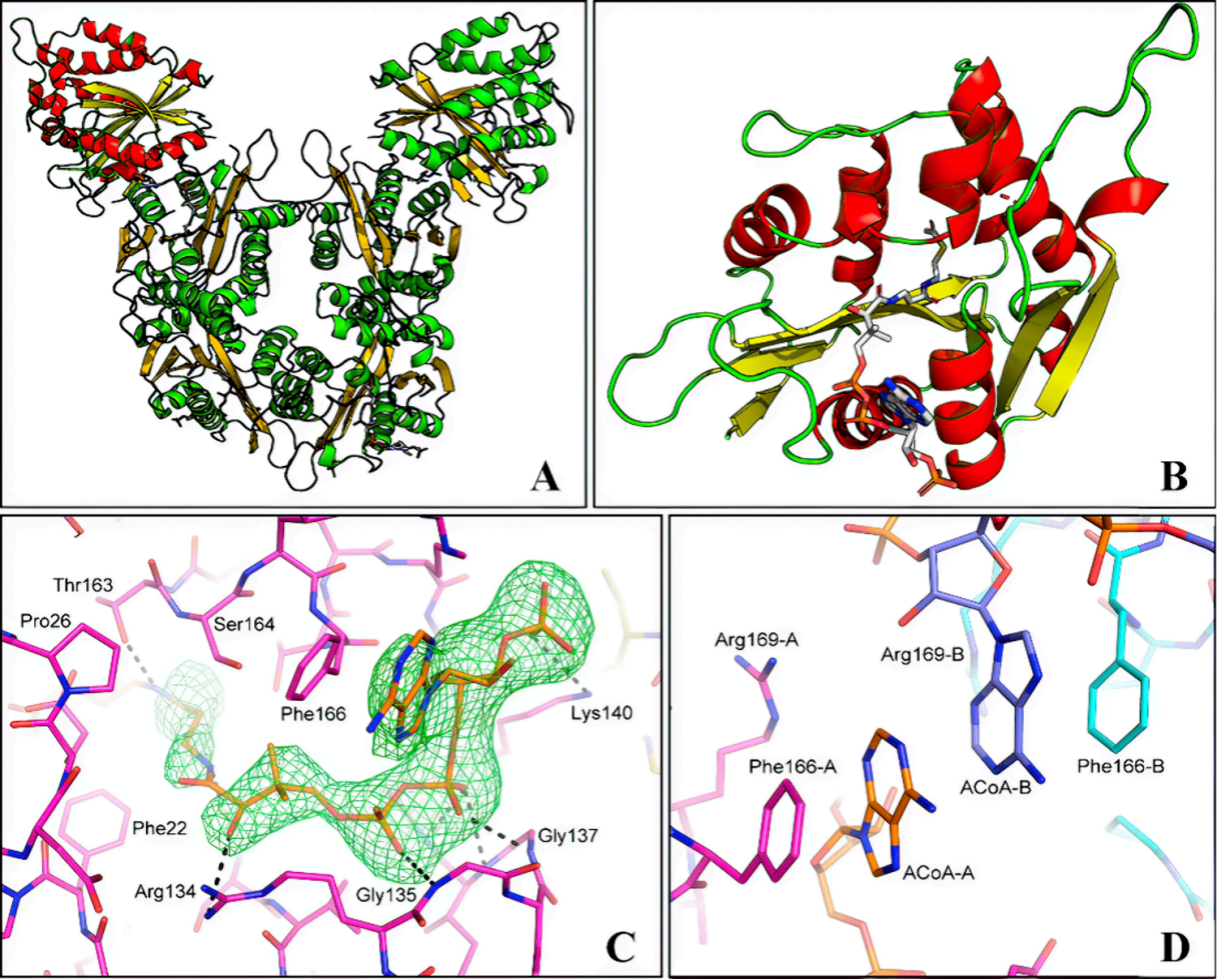
High-resolution crystal structure of TcAANAT0 in complex with acetyl-CoA at a 2.84 Å resolution. (a) The asymmetric unit reveals the hexameric arrangement of TcAANAT0 in the crystal lattice. (b) A single TcAANAT0 monomer is shown with bound acetyl-CoA, illustrating the enzyme–substrate complex. (c) Close-up view of the TcAANAT0 active site with acetyl-CoA bound. The unbiased Fo–Fc electron density map, contoured at 3σ and shown in green, confirms the position of acetyl-CoA within the active site. TcAANAT0 is depicted in magenta, acetyl-CoA in orange, and hydrogen bonds are indicated by black dashed lines. (d) An intermolecular π-stacking interaction is observed between the acetyl-CoA molecule and Phe-166 residue across two adjacent TcAANAT0 molecules at the crystal packing interface. The two interacting TcAANAT0 molecules are colored in magenta and teal, while the acetyl-CoA molecules are shown in orange and blue. Reprinted with permission from ref (98). Copyright 2020 American Chemical Society.

The judicious and precise application of pesticides, targeting specific pests or areas, represents a vital element within IPM approaches, which emphasizes the strategic deployment of chemical control measures.^5^ Recent advancements in research have paved the way for the implementation of precise and focused pesticide application techniques. This approach necessitates a thorough insight into the pest life cycle, ecological interactions, and population fluctuations, as well as the crop phenology and the complex relationships within agricultural ecosystems.^96,97^ Molecular studies have significantly contributed to this endeavor by shedding light on the underlying mechanisms that determine the selectivity of insecticides. For instance, O’Flynn et al.^98^ characterized arylalkylamine N-acyltransferase from *Tribolium castaneum* (TcAANAT0) as a potential insecticide target (Figure 2). Kinetic analysis revealed that short-chain acyl-CoAs (C2–C10) and various arylalkylamines served as substrates, with catalytic efficiencies (kcat/KM) ranging from 1.0 × 103 to 1.2 × 106 M^–1^ s^–1^. The kinetic mechanism was determined to be a sequential mechanism starting with acetyl-CoA binding first, as evidenced by dead-end inhibition patterns and isothermal calorimetry (*K*_d_ = 0.33 ± 0.04 μM for acetyl-CoA). pH-rate profiles and kinetic studies with a novel amine substrate, 2,2-difluoro-2-phenethylamine, provided insight into the chemical mechanism, suggesting that an active site base deprotonates the amine substrate prior to nucleophilic attack. These findings offer valuable structural/mechanistic information for the rational design of inhibitors targeting TcAANAT0 and other insect AANATs.

Lu et al.^99^ discovered lynamicin B as a potent and selective inhibitor for OfChi-h, a group h Chitinase obtained from the lepidopteran pest (i.e., *Ostrinia furnacalis*). Kinetic studies revealed that lynamicin B competitively inhibits OfChi-h with a *K*_i_ of ∼9 μM, while not significantly inhibiting other chitinases. Feeding experiments revealed that lynamicin B displayed potent insecticidal activities against lepidopteran pests *Mythimna separata*, *O*. *furnacalis*, and *Spodoptera frugiperda*, with LC50 values ranging from 20.0 to 53.1 mg/L. These findings suggest that lynamicin B is an intriguing natural-derived pesticide for the control of lepidopteran pests, with minimal impact on nontarget beneficial insects.

Shen et al.^100^ designed, produced, and assessed an array of glycosylated naphthalimide derivatives as novel inhibitors of OfHex1, an insect β-N-acetylhexosaminidase from the agricultural pest *Ostrinia furnacalis*. Rational molecular design and optimization led to the discovery of compounds 15r and 15y, which exhibited potent inhibitory activity against OfHex1 with *K*_i_ values of 5.3 μM and 2.7 μM, respectively, surpassing the efficacy of previously reported lead compounds. In vivo bioassays revealed that the most potent OfHex1 inhibitors exhibited insecticidal activity against *Plutella xylostella*, *Myzus persicae*, and *O. furnacalis*, with compound 15y causing 70% mortality in O. *furnacalis* larvae at 1 mM after 20 days.

Samurkas et al.^101^ virtually conducted a structure-based screening targeting the intersubunit interface of the diamondback moth (DBM) ryanodine receptor (RyR) N-terminal domain (NTD) to identify potential species-specific insecticides. Binding mode analysis revealed that these compounds selectively bind to a hydrophobic region of the DBM NTD-A but not to the corresponding region of its mammalian equivalent. These compounds were tested on HEK293 cell lines stably expressing DBM or mammalian RyR, with one compound showing good potency (EC50 = 58.2 ± 7.6 μM) and selectivity (selectivity index >8.6) against the DBM RyR. The insecticidal effect of this compound was further assured via fruit flies, with an LD50 of 2.51 μg/g. This study presents a baseline for designing a new class of selective RyR-targeting insecticides to manage both wild-type and resistant pests.

#### II.D.2 Resistance Management Strategies (RMS)

RMSs aim to prevent or delay the buildup of pesticide resistance in target pest populations.^102,103^ The facilitation of resistance is driven by the selection stress exerted by repeated pesticide applications, which favor the survival and reproduction of resistant individuals over susceptible ones. RMSs are formulated to mitigate the selection stress exerted on pest populations and to maintain the long term efficacy of pesticides.^104,105^ The alternation of pesticides with different ways of action diminishes the selection pressure on specific resistance mechanisms and aids in maintaining a diverse genetic pool of susceptible individuals within the pest population. Applying pesticides at their full recommended doses constitutes another crucial RMS, as sublethal doses can facilitate the survival and breeding of resistant individuals, thereby accelerating the appearance of resistance.^106^

**Figure 3:**
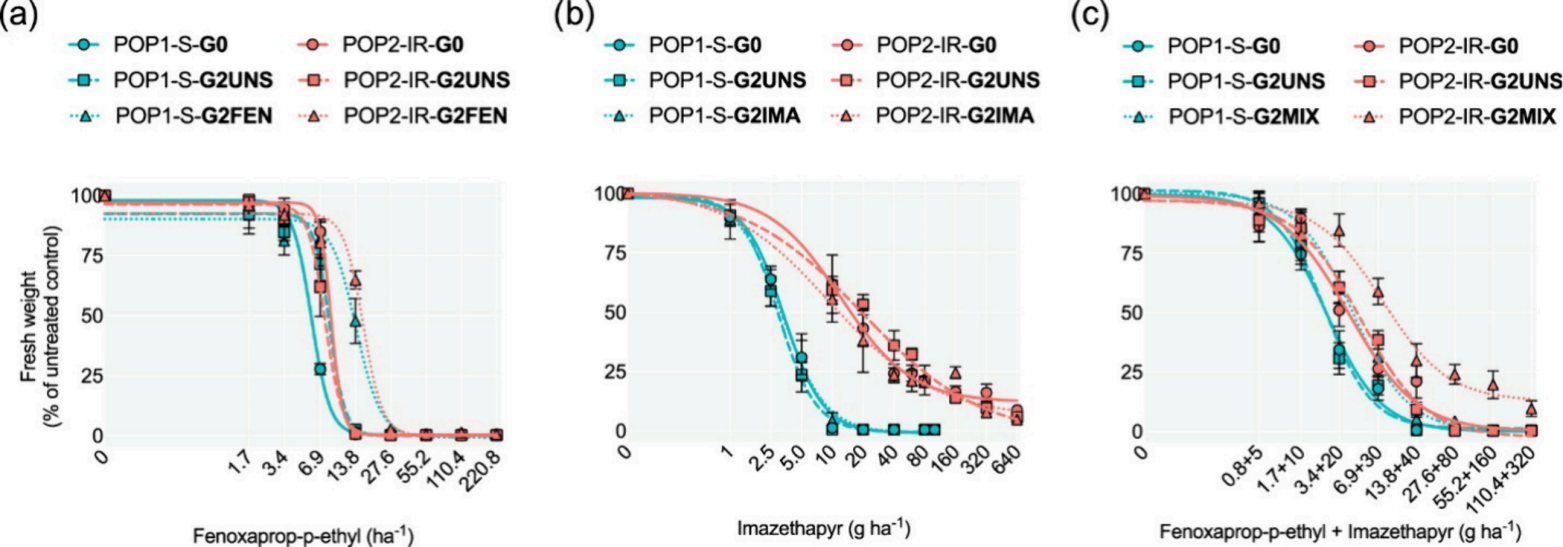
Dose–response curves illustrating herbicide resistance evolution in *Echinochloa crus-galli* (barnyard grass) populations after two generations of low-dose herbicide selection. Curves represent shoot fresh weight (% of untreated control) for susceptible (POP1-S, blue) and initially resistant (POP2-IR, red) biotypes treated with (a) fenoxaprop-*p*-ethyl, (b) imazethapyr, and (c) a mixture of both herbicides. Shifts in curve positions between generations indicate rapid evolution of resistance profiles under sublethal herbicide exposure. Reprinted with permission from ref (110). Copyright 2023 Elsevier.

There are various relevant studies to pest resistance development and management strategies in the literature.^107−109^ Among many studies, a very recent study by Rigon et al.^110^ is particularly noteworthy. Their research investigated how fenoxaprop-*p*-ethyl and imazethapyr herbicide mixture influences the progression of herbicide resistance in *Echinochloa crus-galli* using recurrent selection at sublethal doses (Figure 3). Second-generation offspring chosen with the mixture exhibited reduced pest control efficacy compared to both the parental generation and unselected progeny. Following two selection cycles with the mixture, the GR50 values of susceptible (POP1-S) and imazethapyr-resistant (POP2-IR) biotypes increased by 1.6-fold and 2.6-fold, respectively. This study demonstrated that recurrent selection with a sublethal mixture of herbicides could potentially promote the progression of cross-resistance to cyhalofop, sethoxydim, diclofop, and quinclorac.

#### II.D.3 Biopesticides and Naturally Derived Products

Biopesticides and naturally derived products offer more environmentally benign and sustainable alternatives to conventional synthetic pesticides.^111,112^ Naturally derived products are extracted or isolated from natural materials and may undergo some chemical modification to enhance their efficacy or stability.^113,114^ Microbial pesticides originate from bacteria, fungi, viruses, and nematodes that are pathogenic to specific pest species.^115^ Examples include *Bacillus thuringiensis* (Bt) insecticides, which contain bacterial spores and crystal proteins that are toxic to certain lepidopteran, coleopteran, and dipteran pests,^116^ and entomopathogenic fungi such as *Beauveria bassiana* and *Metarhizium anisopliae*, which infect and kill many arthropod pests.^117^ Likewise, various essential oil-based formulations have been demonstrated to inactive bacterial pests related to food security/safety.^118−124^

R&D efforts in this area center on the discovery and characterization of new bioactive compounds from natural sources and the optimization of formulation and delivery systems. For instance, a recent study investigated the influence of romidepsin to the herbicidal action of the biopesticide *Burkholderia rinojensis*.^125^ It was determined that romidepsin is a biological proherbicide that aims at plant histone deacetylases (HDAC). Romidepsin’s biological activity was significantly enhanced upon reduction of its disulfide bridge by tris(2-carboxyethyl)phosphine hydrochloride (200 mM), resulting in the release of a substantially reactive free butenyl thiol terminal groups. This bioactivation process, involving disulfide bridge reduction, was also observed in plant cell-free extracts.

In a recent study, the phytotoxicity and entomotoxicity of rosemary and artemisia essential oils (EOs) were evaluated against the tomato pest *Bemisia tabaci* when formulated as atomized powders, nanoemulsions, and Natural Deep Eutectic Solvents (NaDES) compared to pure EOs.^126^ Nanoemulsions were reported to have the most entomotoxicity, inducing 60% and 98% mortality for Artemisia and Rosemary EOs, respectively, followed by NaDES (14% and 96% mortality) and pure EOs (17% and 90% mortality), while atomized powders showed no significant entomotoxicity. The extent of plant damage exhibited a comparable pattern, with nanoemulsions inducing the most severe phytotoxic effects, while natural deep eutectic solvents (NaDES) mitigated the deleterious impact on plants in comparison to the application of unmodified essential oils (EOs).

**Figure 4:**
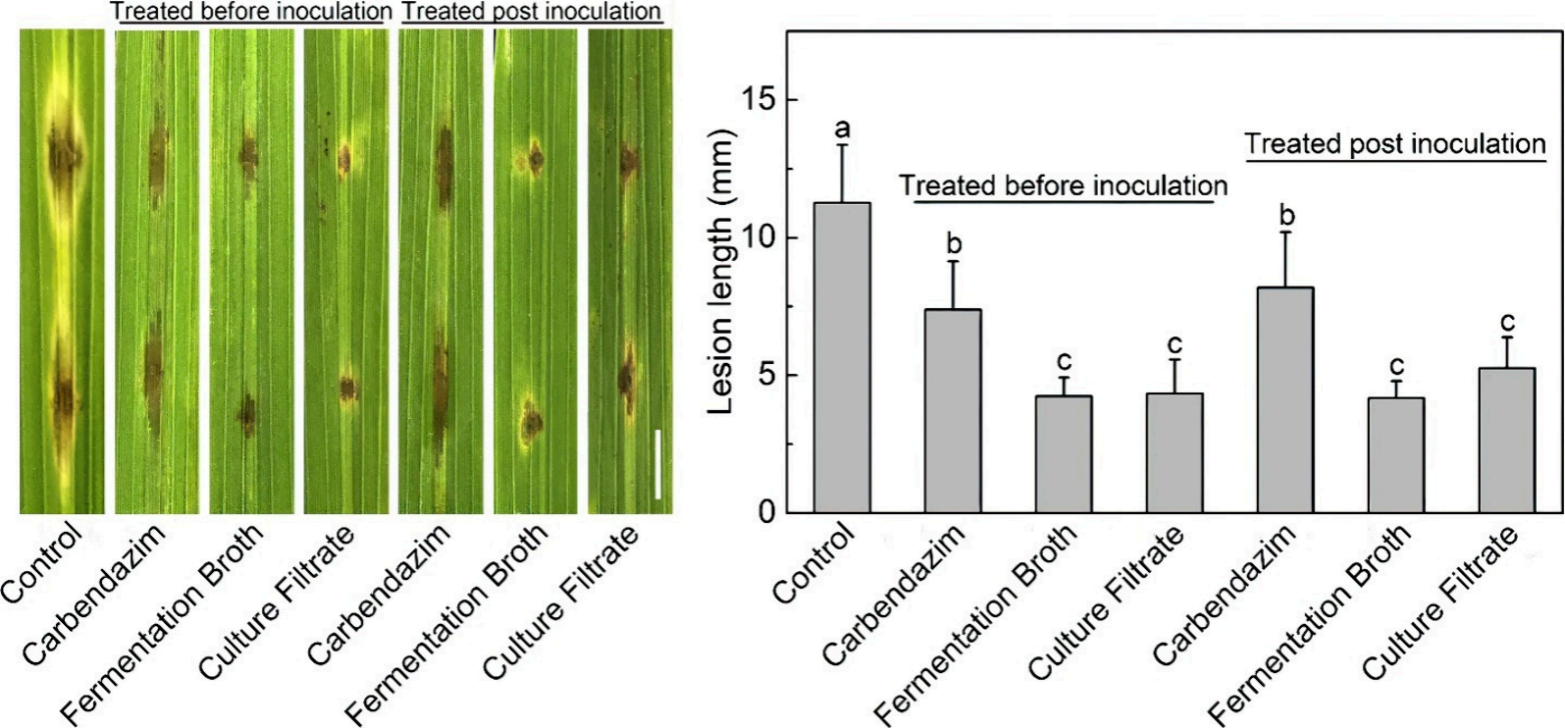
Progression of lesion diameter 5 days postinoculation with strain B21 under preventative and curative droplet treatment regimens. Error bars indicate standard deviation. Statistically significant differences (*p* < 0.05) between treatments are denoted by different letters. Scale bar represents 10 mm. Reprinted with permission from ref (127). Copyright 2020 Elsevier.

Rong et al.^127^ investigated the antifungal activity of the endophytic bacterium *Bacillus safensis* B21 against the rice blast pathogen Magnaporthe oryzae. The extract (with methanol) of B. safensis B21 culture filtrate exhibited strong, dose-dependent inhibition of *M. oryzae* growth (IC50 = 15.56 μg/mL) and remained stable across a wide pH range (1–9) and at temperatures up to 100 °C. In detached leaf assays and field trials, fermentation broth and culture filtrate of *B. safensis* B21 outperformed the fungicide carbendazim in controlling rice blast disease when applied preventatively (Figure 4). The antifungal compounds were detected as the cyclic lipopeptides iturin A2 and iturin A6, which caused morphological abnormalities and increased membrane permeability in *M. oryzae* hyphae.

Giunti et al.^128^ investigated the repellency and habituation effects of essential oil (EO)-based nanoemulsions against the lesser grain borer, *Rhyzopertha dominica*. Stable nanoemulsions were developed using fennel, mint, and sweet orange EOs, with droplet sizes smaller than 200 nm. All EO-based nanoformulations exhibited repellency against *R. dominica* in both area choice and arena bioassays, with mint and sweet orange formulations showing the strongest effects. Habituation to the repellent effects was observed in *R. dominica* adults following successive exposures to mint and sweet orange nanoemulsions, with the decline in responsiveness being frequency-dependent. Complete recovery of repellency was observed 24 h after the last training session.

Citronella grass (*Cymbopogon winterianus Jowitt*) essential oil (EO) and its nanoemulsion were evaluated for their insecticidal and antifeedant activities against the destructive agricultural pest Spodoptera litura.^129^ Citronella essential oil (EO), characterized by GC-MS, revealed 13 terpenoids, with citronellal (26.38%), trans-geraniol (24.61%), and citronellol (13.80%) as the major constituents. A stable oil-in-water (O/W) nanoemulsion was formulated using 15% citronella EO and 5% Tween 80. Laboratory bioassays demonstrated 100% mortality of *S. litura* larvae at concentrations of 10.0 and 12.50 mg/mL for citronella EO and its nanoemulsion, respectively.

#### II.D.4 Nanotechnology

Nanotechnology is an emerging field that holds promise for the design of novel and improved tools for chemical control under IPM.^130^ Nanopesticides offer several potential advantages over conventional pesticide formulations, including increased efficacy, reduced environmental effects, and targeted delivery to predetermined pests or plant tissues.^131−133^ For example, nanoencapsulation of pesticide active ingredients can improve their stability, solubility, and controlled release, thereby lowering the amount of pesticide needed and minimizing off-target effects.^134−136^ Nanoformulations can also enhance the penetration and translocation of pesticides within plant tissues, allowing for more efficient and localized pest control.^137−139^ Examples of nanomaterials employed in the preparation of nanopesticides include polymeric nanoparticles, lipid-based nanocarriers, and inorganic nanoparticles such as silica and titanium dioxide.^140−145^

**Figure 5:**
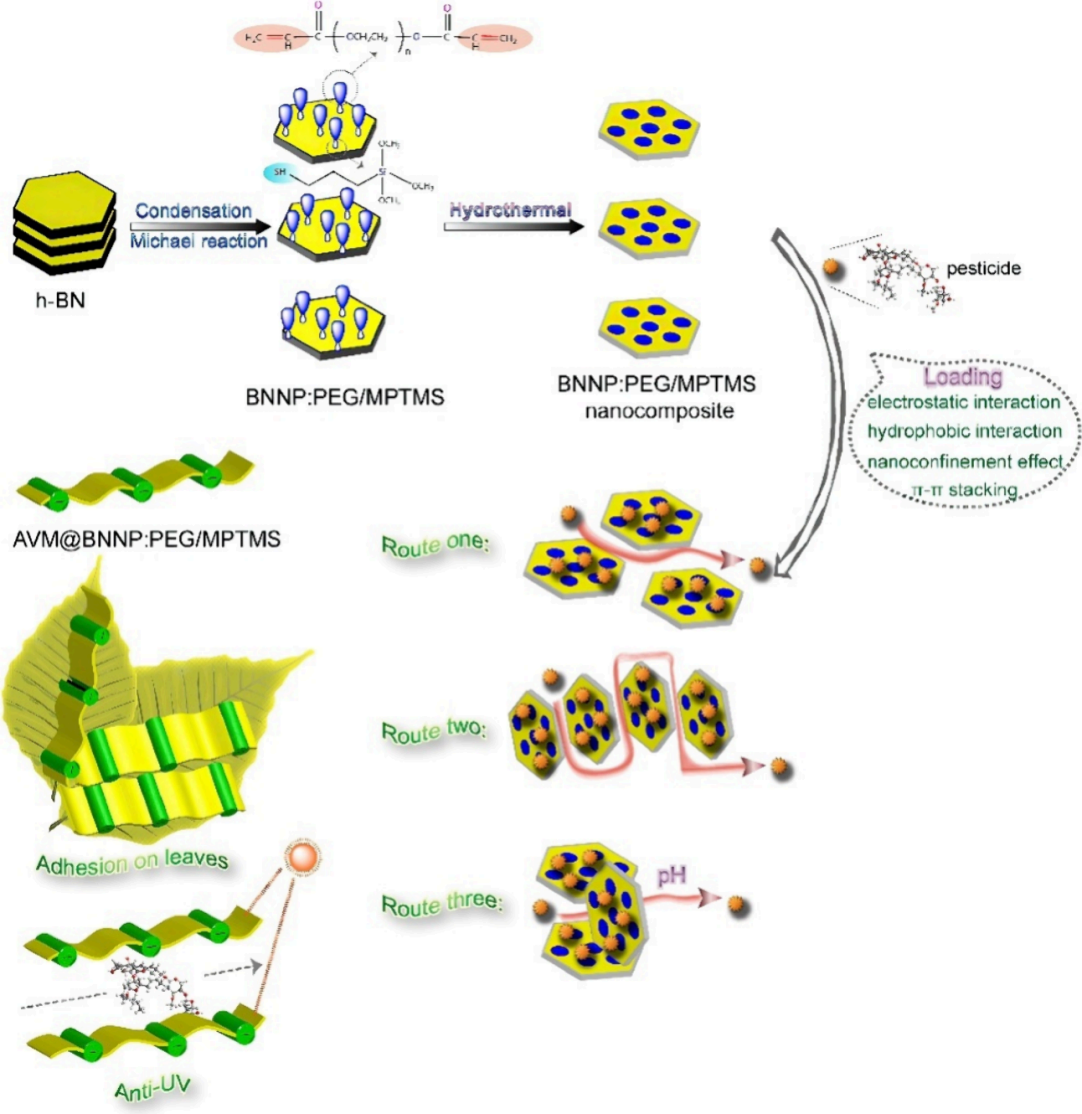
Schematic representation of BNNP functionalization with MPTMS and PEG to create BNNP:PEG/MPTMS composite nanocarriers for pesticide delivery. These nanocarriers exhibit high loading capacity, pH-responsive controlled release, enhanced leaf adhesion, and UV-blocking properties. Functionalized BNNPs self-assemble into interconnected nanochannels with three distinct orientations: horizontally stacked parallel channels vertically stacked tortuous channels and inclined stacked twisted channels. These diverse nanochannel configurations facilitate pesticide release and transport through multiple pathways. Reprinted with permission from ref (146). Copyright 2020 Elsevier.

The development and treatment of nanopesticides in IPM require a multidisciplinary approach that combines expertise from fields such as chemistry, materials science, agronomy, toxicology, risk assessors, regulator, and social sciences. Ongoing research priorities and activities in this area include the design and synthesis of novel nanomaterials with specific functionalities, the optimization of nanoformulations and delivery methods, and the assessment of their efficacy, safety, and environmental fate. For instance, Hao et al.^146^ reported a novel nanopesticide system involving boron nitride nanoplatelets (BNNP) functionalized with polyethylene glycol (PEG) and 3-mercaptopropyl trimethoxysilane (MPTMS) as nanocarriers for the pesticide avermectin (AVM) (Figure 5). They found that the resultant BNNP:PEG/MPTMS nanocomposite exhibited a high pesticide loading capacity of 181.91 ± 5.22 mg/g, attributed to the favorable hydrophobic interactions, π–π stacking, and electrostatic interactions between the nanocarriers and AVM. The presence of PEG on the nanocarrier surface enhanced the colloidal stability and water dispersibility of the nanopesticide. Notably, the release kinetics of AVM from the nanocarriers could be tuned from first-order to zero-order by increasing the pH from 7 to 11, with a two-to-3-fold increase in the release rate, due to the hydrolysis of PEG ester groups under alkaline conditions. Furthermore, the BNNP:PEG/MPTMS nanocarriers significantly improved the photostability of AVM against UV degradation, extending its half-life from 53 to 130 min under UV exposure.

Bae et al.^147^ developed a novel biopesticide nanocomposite encapsulating azadirachtin, a natural insect-killing compound from neem seed, using whey protein isolate as a nanocarrier matrix. The 260.9 ± 6.8 nm nanocomposite demonstrated faster action and greater efficacy against the fall armyworm compared to bulk azadirachtin, with LC50 values determined within 11 days of larval survival. Confocal microscopy revealed enhanced biodistribution throughout the insect body, and the nanocomposite exhibited improved UV stability owing to its intrinsic nanostructure and UV-scavenging vitamin E. This advancement in sustainable pest management highlights the potential for more environmentally friendly approaches to controlling agricultural pests via the combination of biotechnology and nanotechnology.

The study by Zheng et al.^148^ reported the propensity of using chitosan-based nanoformulations to boost the efficacy and sustainability of chlorfenapyr (CHL) for controlling *Spodoptera frugiperda* in maize. They found that CHL was successfully encapsulated in chitosan/carboxymethyl chitosan nanoparticles (CHL@CS/CMCS NPs) with a particle size of approximately 100 nm, which significantly improved the systemic activity of CHL. Root irrigation with CHL@CS/CMCS NPs yielded an optimal efficacy of 89.46–92.36% against *S. frugiperda* 7 days postapplication, which was 7.5–17.5 times higher than that of the CHL suspension concentrate (CHL-SC). The nanoformulation maintained 39.08–65.21% efficacy even 20 days after application. CHL@CS/CMCS NPs were readily absorbed by maize roots and predominantly transported to tender leaves, the preferred feeding site of *S. frugiperda* larvae, enabling targeted delivery of the insecticide.

## III Sustainability Benefits of IPM

Since the initial studies of IPM by Juan Herrera in cotton crops in 1956 in Canete, Peru,^149^ to its confirmation in alfalfa crops in 1959 in California^150^ and later systematization of the IPM concept by Ray Smith in the 1960s,^151,152^ the big challenge has been its generalized systematic and upscale implementation.^153^ However, it was not until the mid-1990s that successful IPM systematic and upscale implementations led by Fausto Cisneros in a range of crops including potatoes, sweet potatoes, asparagus was achieve by use of the Pilot Unit strategies.^154,155^ These were some of the pioneer works on the upscale implementation of sustainability benefits of IPM programs. After this historical synopsis, herein we discuss the most recent advances in sustainable environmental, economic and social benefits of IPM.

### III.A Environmental Sustainability

By prioritizing nonchemical methods and judicious pesticide application based on economic thresholds and pest monitoring, IPM seeks to maintain pest populaces below economically damaging levels while minimizing reliance on chemical interventions. This approach can not only reduce the total volume of pesticides applied but also promote the employment of more selective and benign compounds, mitigating the detrimental influences on nontarget organisms, ecosystems, and human health. IPM employs a combined approach, mixing cultural, biological, and physical control tactics, complemented by the strategic application of reduced-risk pesticides (i.e., biopesticides and naturally derived products). These alternatives, including microbial insecticides, botanical extracts, and semiochemicals, exhibit lower toxicity, shorter persistence, and fewer nontarget effects compared to typical synthetic pesticides. Their incorporation into IPM programs can advance the overall sustainability of crop protection approaches by reducing environmental contamination risks, protecting natural enemies and wildlife, and promoting ecosystem resilience.

Biodiversity, encompassing the variety of life forms at genetic, species, and ecosystem levels, is important for the functioning and resilience of agroecosystems, providing essential services such as pollination, pest control, nutrient cycling, and soil formation.^17,156^ The concentrated use of broad-spectrum pesticides in conventional agriculture has been a major driver of biodiversity loss, disrupting ecological interactions and processes.^157^ IPM promotes biodiversity conservation by prioritizing nonchemical pest control methods and selective pesticide application, mitigating direct and indirect effects on nontarget species. The embracing of cultural practices creates diverse habitats that support various beneficial organisms, enhancing natural pest regulation and overall agroecosystem resilience.^158,159^ IPM also emphasizes the conservation and augmentation of indigenous natural enemy populations through the provision of alternative food sources, sheltering habitats, and overwintering sites, reducing the need for chemical interventions.

### III.B Economic Sustainability

IPM offers a cost-effective approach to crop protection by optimizing pest control through a reduced reliance on relatively expensive and potentially hazardous chemical inputs. The economic gains of IPM arise from reduced pest management costs, improved resource utilization efficiency, and enhanced profitability and competitiveness of agricultural production.^160^ By considering various factors and utilizing available knowledge, IPM allows farmers to carefully evaluate the economic, environmental, and social implications of various pest management techniques. Through the embracing of IPM, farmers can optimize the long-term viability and adaptability of their agroecosystems, while minimizing potential risks and costs associated with pest control interventions. Ultimately, IPM represents a holistic pest management framework, that empowers farmers to make data-driven decisions that prioritize the cumulative health and resilience of their farming systems.^161,162^

The judicious employment of pesticides, informed by economic thresholds, pest monitoring, and decision support systems, can significantly reduce the quantity of chemicals needed to keep pest populations below damaging levels, lowering input costs for farmers and mitigating the development of pesticide resistance. Alternative pest management (e.g., cultural control, biological control) provide cost-effective alternatives to chemical control. IPM also improves the economic efficiency of agricultural production by optimizing the employment of resources like land, water, and labor through precision farming techniques and integration with other sustainable agricultural practices. Crop losses attributable to pests constitute a major constraint to agricultural throughput, with estimates of up to 40% of global crop production lost annually to pests.^163^ The effectiveness of IPM in reducing crop losses has been showcased in numerous studies across different crops and regions. For instance, Hutchison et al.^164^ reported that the widespread adoption of transgenic Bt maize in the U.S. significantly suppressed European corn borer populations across large areas. Their economic analysis estimated that this has provided a cumulative benefit of 6.8 billion USD over 14 years to maize growers in five major maize producing states, with a substantial portion (4.3 billion USD) accruing to farmers growing non-Bt maize.^164^

A pioneer successful approach in applying systematically and upscaling IPM programs was through the use of the Pilot Unit strategies proposed and led by Cisneros,^155^ which worked independently of the type of land tenure and political regime at the time of its application. For instance the use of Pilot Unit strategies in highland Andean quechua cooperative communities decreased damages caused by the crisis of the Andean potato borer beetle *Premnotrypes spp* from ∼45% to 4% in the mid-1990s^155^ and still in use throughout the Andean region. Similarly, the Pilot Unit strategy was applied during the Cuban sweet potato (boniato) crisis under a land tenure government owned communist regime, reducing damage caused by the beetle *Cylas formicarius* (Fab.) from ∼50% to <5% in the mid-1990s^165^ and extensively used in Cuba at present time covering most of its productive land. Finally, this same Pilot Unit approach was applied successfully in the asparagus crisis in the early 2000s in the exporting fields of Chavimochic in Peru owned by large land tenure private companies. These lands were gained to the desert through drip irrigation systems that initially generated ecological imbalances exacerbated by the indiscriminate use of synthetic pesticides, with large damages caused by different pests including *Prodiplosis longifila*, *Bemisia argentifolii, Spodoptera ochrea*, *Pseudoplusia includens*, and *Heliothis virescens*.and reversed by the above IPM strategy, reducing costs of pesticide application from ∼1,200 USD per hectare to 300 USD and applied in ∼3,800 ha by 2003^155^ and extending its use to 7,000 ha of Asparagus for exports by 2015 representing 147 million USD in exports. At present time Chavimochic is the largest exporting area of Peru and one of the largest in the world with exporting crops including avocados, grapes, blueberries, citrus, artichokes, peppers, onions among others, most of which have adapted IPM strategies as those initially applied to asparagus.

### III.C Social Sustainability

IPM can partake in social sustainability by improving food safety and quality, which are essential aspects of human health and well-being. IPM practices prioritize the utilization of nonchemical pest control methods and the judicious use of pesticides, thereby reducing the potential for pesticide residues in food and the related risks to consumer health. Moreover, by curtailing the damage caused by pests and diseases, IPM can help to maintain the nutritional value, appearance, and shelf life of agricultural products, further enhancing their quality and marketability.

Aside from reducing pesticide residues, IPM can also enhance food safety by minimalizing the risk of foodborne illnesses associated with microbial contamination. By endorsing the adoption of good agricultural practices, including proper sanitation, worker hygiene, smart surfaces, and postharvest handling, IPM can aid in preventing the introduction and dissemination of pathogens throughout the food supply chain.^166−170^ Moreover, the utilization of biopesticides within the IPM framework can alleviate the occurrences of harmful chemicals that may interact with foodborne pathogens and exacerbate their virulence or resistance to antimicrobial treatments.^171,172^

IPM is important in lowering the exposure of farmers and consumers to pesticides, which is a key aspect of social sustainability in agriculture. Pesticide exposure can have significant harmful consequences on human health, ranging from acute poisoning to chronic diseases such as neurological disorders, cancer, and reproductive problems.^173,174^ Moreover, the health risks linked to pesticide use are often disproportionately borne by vulnerable populations, such as smallholder farmers, rural communities, and developing countries, where access to protective equipment, training, and health care may be limited.^175,176^ IPM mitigates these challenges by curtailing dependence on chemical pesticides and prioritizing the implementation of safer and more sustainable pest control methods.

## V Challenges and Opportunities

Despite the well-documented values of IPM for environmental, economic, and social sustainability, its prevalent adoption in agricultural systems remains a substantial challenge. Various barriers, including technical, economic, institutional, and cultural factors, can hinder the successful embracing of IPM practices by farmers and other stakeholders. Identifying and addressing these barriers is crucial for promoting the wider implementation of IPM and realizing its promise for sustainable crop protection.

A key technical barrier to IPM adoption is the inherent complexity and knowledge-intensive nature of IPM practices, which necessitates a significant investment in education, experimentation, and adaptation by farmers.^3^ To overcome this barrier, it is imperative to develop and disseminate IPM knowledge and skills through participatory and farmer-centered approaches, such as farmer field visits, on-farm demonstrations, and peer-to-peer learning networks. Moreover, the integration of traditional and local knowledge with scientific research can contribute to the development of more relevant and acceptable IPM strategies tailored to diverse agroecological and socio-cultural contexts.

Economic barriers, including the higher initial costs and perceived risks associated with IPM adoption, can also limit the uptake of IPM practices by farmers.^12,177^ The advantages of IPM might not be immediately apparent or may be subject to uncertainty and variability, depending upon the specific crop, pest complex, and market conditions.^178^ To address these economic barriers, it is important to construct and implement policies and incentives that support the adoption of IPM practices, such as subsidies, credits, and market-based instruments. For example, the European Union’s Common Agricultural Policy (CAP) provides agri-environment payments to farmers who adopt IPM and other sustainable farming practices, recognizing their contribution to public goods and ecosystem services.^179,180^ Similarly, the development of value chains and certification schemes for IPM-based products can create market incentives for farmers to adopt IPM practices and differentiate their products from conventionally grown ones.

Cultural and social barriers, such as the perception of IPM as a complex and risky approach, can also limit the adoption of IPM practices by farmers.^181^ In many cases, farmers may be reluctant to change their established pest management practices, especially if they perceive IPM as a threat to their identity, autonomy, or social status.^182^ To address these cultural and social barriers, it is essential to engage farmers and other stakeholders in the codesign and coimplementation of IPM strategies, considering their knowledge, values, and aspirations. Participatory and empowering engagement approaches, such as community-based IPM, can help to build trust, reciprocity, and collective action among farmers, while also fostering a sense of pride and ownership in the adoption of IPM practices.^183^ For instance, the promotion of the successful IPM program based on a Pilot Unit strategy described earlier for systematic and upscale IPM implementation could be an answer to these challenges including different crops, land tenure type and government regime.^155,184^

IPM is not a standalone approach but rather an integral component of sustainable agricultural systems that aim to optimize the utilization of natural resources, enhance ecosystem services, and improve the resilience and adaptability of agroecosystems.^185^ The integration of IPM with other sustainable agricultural practices, such as conservation agriculture, agroforestry, and organic farming, can create synergies and cobenefits that enhance the overall sustainability and performance of agricultural systems. Moreover, the incorporation of IPM with broader sustainable development goals, such as biodiversity conservation, climate change mitigation and adaptation, and rural livelihood improvement, can help to scale up and mainstream IPM practices in different agroecological and socio-economic contexts.^186^ However, integrating IPM with other sustainable agricultural practices and broader development goals also presents challenges. These include the necessity for cross-sectoral coordination and collaboration, the development of context-specific knowledge and solutions, and the foundation of enabling policies and institutional frameworks.

Labor constraints present another challenge for IPM adoption as the agricultural sector is facing increasing labor shortages and rising labor costs. IPM often requires more intensive monitoring, scouting, and management practices compared to conventional pest control methods. This increased labor demand can be a substantial barrier for growers already struggling to find and afford workers. Another practical challenge is the limitations of biopesticides. While biopesticides are an important tool in IPM, relying solely on them presents several issues. Biopesticides are often more expensive than conventional pesticides and typically require higher application rates. They also need more frequent applications. Moreover, biopesticides tend to provide only partial pest suppression rather than complete control. These factors can make exclusive reliance on biopesticides economically unfeasible for many growers, necessitating a more balanced approach that may still include some use of conventional pesticides within an IPM framework.

The regulatory milieu surrounding IPM varies significantly across major agrarian nations, influencing its implementation trajectory. In the U.S., the Endangered Species Act’s stipulations regarding pesticide application in relation to protected species engender a complex regulatory framework that complicates IPM implementation.^187^ The EU’s evolving phytosanitary legislation has recently precipitated unrest among farmers due to perceived conflicts between ecologically driven policies and agricultural viability. In Brazil, the nexus between agricultural frontier expansion and biodiversity conservation complicates IPM adoption in areas such as the Cerrado and Amazon biomes.^188^ Australia’s unique island biogeography necessitates innovative IPM protocols, yet regulatory frameworks often lag behind scientific advancements in biopesticide development and novel biological control agents. These different regulatory landscapes indicates the imperative for adaptive, context-specific IPM strategies that can navigate the complex issues among agroecosystem management, phytosanitary regulations, and socio-economic exigencies.

## IV Research Gaps and Priorities

Despite the significant progress made in the development and application of IPM strategies over the past decades, there remain important research gaps and priorities that need to be addressed to further enhance the effectiveness, adaptability, and scalability of IPM in different agroecological and socio-economic contexts.

An important research gap in IPM lies in the limited understanding of the complex ecological interactions and dynamics that govern the functioning and resilience of agroecosystems. While IPM has historically focused on managing individual pest species and deploying specific control tactics, there is increasing recognition of the need for a more holistic and systems-based approach. This approach acknowledges the multitude of interacting factors and processes that influence the structure and function of agroecosystems. Key areas of investigation include trophic relationships and food web dynamics among pests, natural enemies, crops and their microbiotas; the role population genetics in modulating perst control effectiveness, the impact of abiotic factors and land use patterns on pest population dynamics; the role of biodiversity and ecosystem preservation in the natural regulation of pests and research of better ways to engage stakeholder communities.

Furthermore, there is a need to increase our understanding of the molecular mechanisms involved in most pest-crop interactions and the evolutionary processes that shape the adaptation and resistance of pests to different control strategies. With the rapid development of genomic and biotechnological tools, there is a growing opportunity to deepen our comprehension of these mechanisms and pathways that likely influence the behavior, physiology, and ecology of pests and their natural enemies. Increasing knowledge in this area will facilitate the development of novel pest control targets. This includes the development of genetically engineered crops, pests, natural enemies and biopesticides that may enhance the efficacy and specificity of pest control.

Despite the significant progress made in the development and application of novel chemical and biological control agents for pest management, there remain important knowledge gaps and research priorities that need to be addressed to further enhance their effectiveness, selectivity, and sustainability. One of the chief knowledge gaps is the limited understanding of the complex interactions and compatibility between different chemical and biological control agents, as well as their impact on nontarget organisms and the broader agroecosystem. While the development of new pesticides and biopesticides with improved plant adsorption and insect targeting properties is important, it is equally crucial to evaluate their potential synergistic or antagonistic effects when used in combination with other control tactics, such as natural enemies, resistant varieties, or cultural practices. This requires a more integrated and systems-based approach that considers the multiple factors and feedback loops that shape the dynamics and consequences of pest control strategies.

Another important research priority is the need for more extensive and rigorous testing and regulation of new chemical and biological control agents, particularly in terms of their postrelease long-term efficacy, safety, and environmental impact. While the use of advanced formulation and delivery technologies, such as nanoemulsions and microcapsules, can enhance the performance and stability of these agents, it is imperative to assess their potential risks and unintended consequences, such as the development of resistance in pest populations, the accumulation of residues in soil and water, or the disruption of ecological processes and food webs. This requires an adaptive approach that incorporates ongoing monitoring, evaluation, and adjustment of pest control stratagems based on the best available scientific evidence and stakeholder feedback. To ensure the responsible development and deployment of these pest control strategies, it is necessary to modernize our current regulatory system for biotech products.

## VII Conclusions

IPM emerged as a promising and sustainable paradigm for crop protection, offering a viable alternative to the excessive and indiscriminate application of chemical pesticides. By synergistically integrating a wide range of preventative, biological, cultural, and chemical control strategies, IPM seeks to keep pest populations below economically damaging thresholds while mitigating risks to public health and the environment. The embracing of IPM practices has been demonstrated to yield multiple benefits, including lowered pesticide use and associated risks, improved crop yields and quality, enhanced biodiversity, and increased profitability and resilience of some farming systems. However, despite the well-documented advantages of IPM, its widespread adoption and scaling have been hindered by various technical, economic, institutional, and social barriers. These barriers include the complexity and knowledge-intensive nature of IPM, the high initial costs and perceived risks of adoption, the lack of supportive policies and market incentives, and the limited awareness and participation of farmers and other stakeholders in the design and implementation of IPM programs. Overcoming these barriers requires a holistic and integrated approach that addresses the multiple dimensions of IPM adoption, and governance from the construction of locally adapted and cost-effective IPM strategies to the creation of enabling environments and value chains and regulations that support the scaling and agricultural-sustainability of IPM. A pioneer and successful approach for systematically and upscale implementation of IPM is the use of the Pilot Unit strategy that can be promoted as a starting point and adapted to a range of crops.

**Figure 6:**
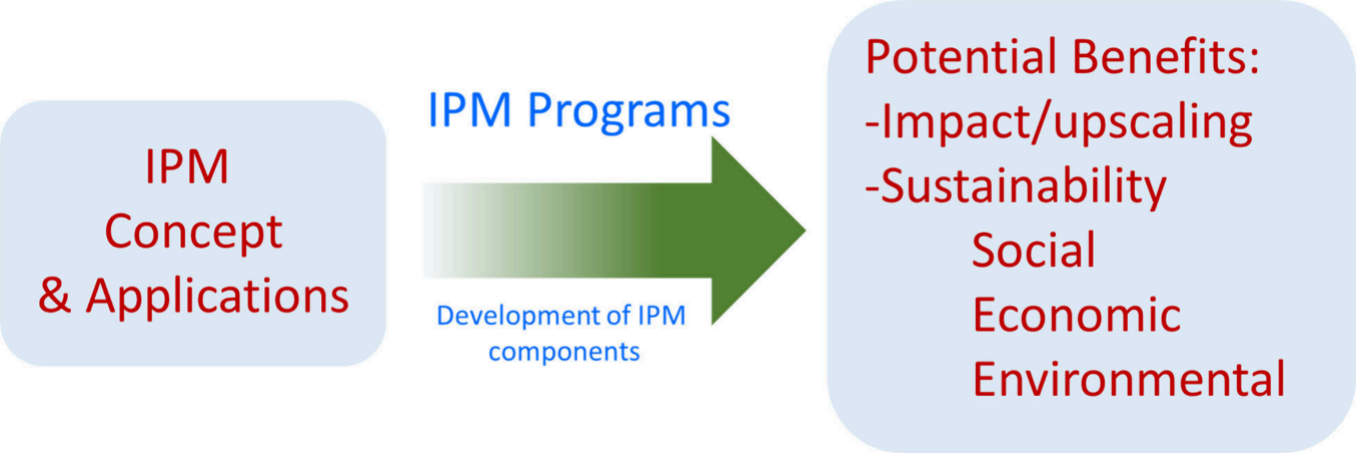
IPM concepts and IPM programs and their potential benefits. For IPM concepts and applications to have an impact in pest control, there is need to develop successful IPM programs that can generate benefits including upscaling and sustainability on the social, economic, and environmental levels. Furthermore, the strength of the IPM programs at the end depends on the strength of the development of novel IPM components.

Moreover, the efficacious and widespread implementation of IPM requires a paradigm shift in the means we approach crop protection and agricultural development, moving from a narrow focus on yield maximization and pest eradication to a more holistic and agroecological perspective that recognizes the complex interactions and trade-offs between productivity, sustainability, and resilience while addressing climate change and sustainability challenges. This shift involves the embracing of IPM with other sustainable agricultural practices, such as conservation agriculture, precision agriculture, agroforestry, and organic farming, as well as the mainstreaming of IPM into broader food systems and landscape management strategies. To achieve this paradigm shift and realize the full potential of IPM, there is a need for increased investment in research, education, and extension that can generate and disseminate knowledge, technologies, and practices that are relevant, accessible, and adaptable to the diverse contexts and needs of farmers and other stakeholders. This includes the development of innovative and participatory research approaches that engage farmers and other stakeholders in the codesign and coevaluation of IPM strategies, as well as the strengthening of the capacity and empowerment of farmers and other actors to adopt and adapt IPM practices to their local conditions. A key player in the successful implementation of an IPM program to generate benefits in pest control that can upscale and be sustainable, is the development of novel IPM components as described in this review (Figure 6).

In conclusion, IPM offers a promising and sustainable approach to crop protection that can contribute to the achievement of the UN Sustainable Development Goals and the transition toward more resilient, inclusive, and sustainable agri-food systems. However, realizing the full potential of IPM requires a concerted and collaborative effort by all stakeholders involved, including farmers, researchers, policymakers, and civil society organizations, to address the multiple challenges and opportunities for the scaling and mainstreaming of IPM. By investing in research, education, and extension that can generate and disseminate relevant and actionable knowledge and practices, and by creating enabling policies and institutions that can support the adoption and diffusion of IPM, we can harness the power of IPM to achieve a more sustainable and equitable future for all.
